# Effects of differently cushioned running shoes at left and right foot on running symmetry

**DOI:** 10.1186/1757-1146-7-S1-A8

**Published:** 2014-04-08

**Authors:** Torsten Brauner, Thorsten Sterzing, Mathias Wulf, Thomas Horstmann

**Affiliations:** 1 Technische Universität München, Munich 80992, Germany; 2 Sports Science Research Center, Li Ning (China) Sports Goods Co Ltd, Beijing 101111, China; 3 Medicalpark St. Hubertus, Bad Wiessee 83707, Germany

## Background

The cushioning of running shoes and leg stiffness influence tibial impact shock [1]. This knowledge, however, is based on investigations with the same cushioning at both feet. Unknown is whether leg stiffness can be adjusted for each leg individually. Thus, the purpose of this study was to quantify effects of differently cushioned running shoes at the left and right foot on running symmetry.

## Methods

Twenty-eighty physically active males (26.8±8.4years, 1.80±0.05m, 74.8±7.5kg), with similar left and right leg stiffness, participated in this study. Two pairs of identical custom-made running shoes, representing harder-cushioned (mechanical impact testing at rearfoot: 13.8g) and softer-cushioned (10.2g) footwear, were used. The four single shoes were combined into four experimental conditions (left foot-right foot): hard-hard, hard-soft, soft-hard, soft-soft). In each condition, subjects ran 200m on a concrete track at self-selected pace. Conditions were blinded, the order randomized and a 100m run was performed in a neutral running shoe between conditions. Directly following each condition, subjects rated the cushioning of the left and right shoe separately on a visual analogue scale (0cm=soft, 10cm=hard). A mobile 3D accelerometer (Humotion, Germany) strapped to the lower back at L5-S1 recorded vertical acceleration. As a measure of running symmetry [2], peak vertical impacts of 32 foot-falls were determined for each leg. Left and right impact peaks and subjective cushioning ratings were compared using paired Student T-Tests (α=.05).

## Results

In both of the mixed conditions, subjects perceived the soft shoe to be significantly softer than the hard shoe (p=.031), according to their actual mechanical impact hardness. Vertical impact peaks at the lower back did not differ between any of the tested conditions and were symmetrical for the mixed conditions.

**Table 1:** Vertical impact at lower back and VAS rating of cushioning perception

	Left hard	Right soft	Left soft	Right hard	Left hard	Right hard	Left soft	Right soft
**Impact [g]**	1.97 (0.50)	2.01 (0.47)	2.01 (0.54)	2.01 (0.49)	2.01 (0.55)	2.02 (0.48)	2.00 (0.52)	2.02 (0.47)
**Rating [VAS 0-10]**	5.1 (2.5)	4.1 (2.2)	4.3 (1.9)	5.0 (2.1)	5.2 (2.3)	5.2 (2.3)	4.6 (2.2)	4.7 (2.2)

## Discussion

Despite the well described effects of shoe cushioning on tibial impact shock, impact at the lower back was not influenced by differently cushioned running shoes. Thus, runners adapted their ankle, knee and/or hip stiffness, reducing the impact shock on its way upward. Interestingly, as runners perceived different cushioning of shoes correctly, this adaptation was controlled for each leg individually, so that also in the mixed cushioning conditions the shock at the lower back remained symmetrical.

## Conclusion

Maintaining low and symmetrical impacts at the lower back seems to be important during running, and is achieved by adjusting the leg stiffness, which can even be controlled for each leg individually. In further research, the mechanism of this individual leg stiffness control should be investigated.
